# Trends in self-reported prevalence and management of hypertension, hypercholesterolemia and diabetes in Swiss adults, 1997-2007

**DOI:** 10.1186/1471-2458-11-114

**Published:** 2011-02-18

**Authors:** Daniel Estoppey, Fred Paccaud, Peter Vollenweider, Pedro Marques-Vidal

**Affiliations:** 1 Institute of Social and Preventive Medicine (IUMSP), University Hospital (CHUV) and Faculty of Biology and Medicine, Bugnon 17, 1005 Lausanne, Switzerland; 2 Department of Medicine, University Hospital (CHUV), Bugnon 21, 1011 Lausanne, Switzerland

## Abstract

**Background:**

Switzerland has a low mortality rate from cardiovascular diseases, but little is known regarding prevalence and management of cardiovascular risk factors (CV RFs: hypertension, hypercholesterolemia and diabetes) in the general population. In this study, we assessed 10-year trends in self-reported prevalence and management of cardiovascular risk factors in Switzerland.

**Methods:**

data from three national health interview surveys conducted between 1997 and 2007 in representative samples of the Swiss adult population (49,261 subjects overall). Self-reported CV RFs prevalence, treatment and control levels were computed. The sample was weighted to match the sex - and age distribution, geographical location and nationality of the entire adult population of Switzerland.

**Results:**

self-reported prevalence of hypertension, hypercholesterolemia and diabetes increased from 22.1%, 11.9% and 3.3% in 1997 to 24.1%, 17.4% and 4.8% in 2007, respectively. Prevalence of self-reported treatment among subjects with CV RFs also increased from 52.1%, 18.5% and 50.0% in 1997 to 60.4%, 38.8% and 53.3% in 2007 for hypertension, hypercholesterolemia and diabetes, respectively. Self-reported control levels increased from 56.4%, 52.9% and 50.0% in 1997 to 80.6%, 75.1% and 53.3% in 2007 for hypertension, hypercholesterolemia and diabetes, respectively. Finally, screening during the last 12 months increased from 84.5%, 86.5% and 87.4% in 1997 to 94.0%, 94.6% and 94.1% in 2007 for hypertension, hypercholesterolemia and diabetes, respectively.

**Conclusion:**

in Switzerland, the prevalences of self-reported hypertension, hypercholesterolemia and diabetes have increased between 1997 and 2007. Management and screening have improved, but further improvements can still be achieved as over one third of subjects with reported CV RFs are not treated.

## Background

Cardiovascular disease is the main cause of premature death in industrialized countries, and its incidence is increasing worldwide [1]. In Switzerland, between 1970 and 2004, mortality rates from ischemic heart and cerebrovascular disease have decreased by circa 50% in men, and by a third by women [2]. Whether those decreases are due to a decrease in cardiovascular risk factors prevalence and/or management is currently unknown.

There are few data regarding trends of cardiovascular risk factors in the Swiss population. The MONICA study showed an increase between 1984 and 1993 in the prevalence of hypertension in men and a decrease in women. For the same time period, a decrease in the prevalence of hypercholesterolemia (defined as a total cholesterol level > 6.5 mmol/L) was also reported for both genders [3]. More recently, data from Geneva showed a decrease in the prevalence of hypertension for both genders between 1993 and 2000. For the same period, an increase in the prevalence of hypercholesterolemia was reported [4]. Still, it is not known if the results of this study also apply to the whole country. Thus, we used the data from the National Health Surveys conducted in representative samples of the Swiss population to assess the trends in self-reported prevalence, treatment and control of hypertension, hypercholesterolemia and diabetes in Switzerland, as well as to identify the groups at higher risk.

## Methods

### Swiss Health Survey

Data from the Swiss Health Surveys (SHS) were obtained from the Swiss Federal Statistical Office (http://www.bfs.admin.ch). The SHS is a cross-sectional, nationwide, population-based telephone survey conducted every 5 years since 1992 (1992, 1997, 2002 and 2007) [5]. The SHS aims to track public health trends in a representative sample of the resident population of Switzerland aged 15 and over.

The study population was chosen by stratified random sampling of a database of all private Swiss households with fixed line telephones. It is currently estimated that over 90% of the Swiss households have fixed telephones. The first sampling stratum consisted of the seven main regions: West "Léman", West-Central "Mittelland", Northwest, Zurich, North-Eastern, Central and South. The second stratum consisted of the cantons, and the number of households drawn was proportional to the population of the canton. In some cantons, oversampling of the households was made to obtain accurate cantonal estimates. Extra strata were used for two large cantons of Zurich and Bern. Within these strata, households were randomly drawn and, within the household, one member was randomly selected within all members aged 15 years and over. A letter inviting this household member to participate in the survey was sent, then contacted by phone and interviewed using computer-assisted software managing both dialling and data collection. The interviews were carried out in German, French or Italian, as appropriate. People who did not speak any of these three languages were excluded from the survey. Other criteria for exclusion were: asylum seeker status, households without a fixed line telephone, very poor health status and living in a nursing home [6]. Four sampling waves were performed (Winter, Spring, Summer and Autumn). Participation rate was 71% in 1992, 85% in 1997, 64% in 2002, and 66% in 2007. More details available at http://www.bfs.admin.ch/bfs/portal/fr/index/infothek/erhebungen__quellen/blank/blank/ess/04.html. As too many data were missing in 1992 (no information on hypertension and diabetes), only data for the three last surveys (1997, 2002 and 2007) was used.

### Data collected

Three age categories were considered: 18 to 44, 45 to 64, and ≥ 65 years. Education was categorized as follows: 1) no education completed, 2) first level (primary school), 3) lower secondary level, 4) upper secondary level and 5) tertiary level, which included university and other forms of education after the secondary level. We defined "low education" (categories 1 and 2), "middle education" (categories 3 and 4), and "high education" (category 5) groups. Self reported height and weight allowed the calculation of Body Mass Index (BMI). Three BMI categories were considered: normal (< 25 kg/m^2^), overweight (≥ 25 to < 30 kg/m^2^) and obese (≥ 30 kg/m^2^). Citizenship was defined as Swiss (having a Swiss passport) or foreigner.

The self-reported prevalence of hypertension, hypercholesterolemia or diabetes was assessed by the questions: "Did a doctor or a health professional tell you that you have high blood pressure/a high cholesterol level/diabetes?", respectively. Subjects were considered as treated for hypertension, hypercholesterolemia or diabetes if they answered positively to the questions "Are you treated for blood pressure/to decrease your cholesterol levels/for diabetes?" respectively. Self-reported prevalence of antihypertensive, hypolipidaemic or antidiabetic treatment was calculated as the ratio of subjects reporting being treated by the number of subjects reporting the disease (i.e. number of subjects reported being treated for hypertension divided by the number of subjects reporting being hypertensive). A further question on doctor-prescribed medicines was asked. All subjects being treated were considered irrespective of the answer to the latter question.

Adequate treatment of hypertension, hypercholesterolemia or diabetes was considered if the subjects answered "normal or too low" to the questions: "Currently, how is your blood pressure/cholesterol level/glycaemia?" respectively. Self-reported prevalence of adequate CV RF management was calculated as the ratio of subjects reporting being treated and answering "normal or too low" divided by the overall number of subjects reporting being treated. Missing answers were considered as negative (i.e. high levels). As the questionnaires changed slightly between surveys, some questions were missing, i.e., the question on control of hypertension was not asked in 2002.

All subjects, irrespective of their status, were asked when they last had their blood pressure, cholesterol or glucose levels measured. Adequate screening was considered if the measurement had been performed during the last 12 months.

### Statistical analysis

Statistical analysis was conducted using Stata version 10 (Statacorp, College Station, TX, USA) and SAS Enterprise Guide version 4.1 (SAS Inc, Cary, NC; USA). Results were expressed as number of subjects and (percentage) or mean ± standard deviation. Comparisons were performed using chi-square for categorical data or analysis of variance (ANOVA) for continuous data. A first analysis was conducted using the original data. A second analysis was conducted after probability weighting each subject according to the formula

$$w_{i}^{h}=H_{i}\cdot \frac{N_{h}}{n_{h}^{n}}$$

Where N_*h *_is the average number of telephone numbers in stratum *h *(h = 29), H_*i *_is the household size, i.e. the number of subjects aged 15 and over living in household *i*, and n_h_^n ^is the number of telephone numbers in the sample S_*h *_corresponding to stratum *h *to the power *n *(*n *= sample size in stratum *h*). Weights were further corrected taking into account the percentage of nonresponders by raking ratio estimation [7]. Weighting partly allowed the correction for bias, i.e. subjects with given characteristics who are under-represented in the original sample were attributed a higher weight [8]. The sum of weights thus corresponds to the Swiss adult population for the period considered. For simplicity, the weighted results will be presented and commented, as the conclusions arising from the unweighted data are similar (see Additional file 1). A third analysis using multivariate logistic regression adjusting for age group, sex, nationality, education and BMI classes was conducted to assess trends during the study period, using either the original (see Additional file 1) or the weighted data (presented here). The results were expressed as Odds ratio and [95% confidence interval]. Statistical significance was considered for p < 0.05.

## Results

### Characteristics of the subjects

The characteristics of subjects according to survey are summarized in table 1. Between 1997 and 2007 mean age increased and the percentage of subjects with low or middle education decreased while the percentage of subjects with high education increased.

**Table 1:** characteristics of the samples

	1997	2002	2007
**Sum of weights**	5,564,776	5,647,472	5,784,057
Women (%)	51.7	51.6	51.2
Age classes (%)			
18-44 years	51.5	49.9	49.5
45-64 years	29.8	30.9	31.9
≥ 65 years	18.7	19.2	18.7
Swiss nationality (%)	81.7	80.5	79.5
Educational level (%)			
Low ^§^	22.6	20.4	12.8
Middle ^§§^	60.0	63.2	59.6
High ^§§§^	17.4	16.4	27.6
BMI classes (%)			
Normal	63.8	61.3	61.0
Overweight	29.1	30.7	30.4
Obese	7.1	8.0	8.6
BMI [kg/m^2^]	24.2 ± 3.9	24.3 ± 4.0	24.4 ± 4.1
Age [years]	46.5 ± 17.6	47.2 ± 17.5	47.3 ± 17.6

Results are expressed as weighted percentage and average ± standard deviation. ^**§ **^no education completed + first level (primary school). ^**§§ **^lower + upper secondary level. ^**§§§ **^tertiary level + other education after secondary level.

### Hypertension

The trends in self-reported prevalence of hypertension are shown in table 2. Between 1997 and 2007, self-reported hypertension in the Swiss general population increased, and this was further confirmed after multivariate adjustment (table 3). Subjects aged over 65 years or obese had a higher odds ratio, while subjects with university level or foreigners had a lower odds ratio of reporting being hypertensive (table 3). Self-reported treatment increased (table 2); on multivariate analysis, subjects aged over 45 or obese had a higher odds ratio, while women and foreigners had a lower odds ratio of reporting being treated (table 3). Self-reported prevalence of treatment prescribed by the doctor was 96.0%, 99.4% and 99.6% while the daily taking of an antihypertensive drug was 89.6%, 95.3% and 97.1% in 1997, 2002 and 2007, respectively. The self-reported prevalence of controlled hypertension increased and the self-reported prevalence of uncontrolled and untreated hypertension decreased (table 2); on multivariate adjustment, subjects over 65 presented a higher odds ratio of reporting being controlled (table 3). Hypertension screening also increased (table 2), and on multivariate analysis, men, foreigners, subjects aged over 45, overweight or obese had a higher odds ratio of being screened (table 3).

**Table 2:** trends in self-reported prevalence and management of hypertension in the Swiss population, 1997 - 2007

	1997	2002	2007
Sum of weights	5,564,776	5,647,472	5,784,057
Hypertension (%)			
Screening	87.7	95.1	95.1
Prevalence	22.1	22.4	24.2
Treatment *	52.1	53.8	60.4
Control **	56.4		80.6

Results are expressed as weighted percentage. *, among subjects reporting being hypertensive; **, among treated subjects. -, data not available.

**Table 3:** multivariate analysis of the trends in self-reported prevalence and management of hypertension in the Swiss population, 1997 - 2007

	Prevalence	Treatment*	Control**	Screening
Surveys				
1997	1 (ref.)	1 (ref.)	1 (ref.)	1 (ref.)
2002	0.98 [0.97 - 0.99]	1.01 [1.01 - 1.02]	-	2.73 [2.71 - 2.74]
2007	1.10 [1.09 - 1.11]	1.32 [1.31 - 1.33]	3.16 [3.13 - 3.18]	2.71 [2.70 - 2.72]
Gender				
Woman	1 (ref.)	1 (ref.)	1 (ref.)	1 (ref.)
Man	1.01 [1.00 - 1.02]	1.17 [1.16 - 1.18]	0.96 [0.95 - 0.96]	0.78 [0.77 - 0.79]
Age groups				
18-44	1 (ref.)	1 (ref.)	1 (ref.)	1 (ref.)
45-64	2.79 [2.78 - 2.80]	4.96 [4.93 - 5.00]	1.89 [1.86 - 1.92]	1.36 [1.35 - 1.37]
≥ 65	7.36 [7.34 - 7.38]	15.1 [15.0 - 15.2]	1.68 [1.66 - 1.71]	2.39 [2.38 - 2.41]
Nationality				
Swiss	1 (ref.)	1 (ref.)	1 (ref.)	1 (ref.)
Other	0.90 [0.89 - 0.91]	0.91 [0.90 - 0.92]	0.56 [0.55 - 0.57]	1.25 [1.24 - 1.26]
Education				
Low	1 (ref.)	1 (ref.)	1 (ref.)	1 (ref.)
Medium	0.93 [0.93 - 0.94]	0.98 [0.97 - 0.99]	1.22 [1.21 - 1.23]	0.95 [0.95 - 0.96]
High	0.90 [0.89 - 0.91]	1.03 [1.02 - 1.04]	1.60 [1.58 - 1.62]	0.83 [0.82 - 0.84]
BMI classes				
Normal	1 (ref.)	1 (ref.)	1 (ref.)	1 (ref.)
Overweight	1.94 [1.93 - 1.95]	1.42 [1.41 - 1.43]	1.12 [1.11 - 1.13]	1.20 [1.19 - 1.21]
Obesity	4.23 [4.22 - 4.25]	1.98 [1.96 - 1.99]	1.07 [1.06 - 1.08]	1.57 [1.56 - 1.59]

Results are expressed as multivariate-adjusted odds ratio and [95% confidence interval]. *, among subjects with reported hypertension; **, among treated subjects. -, data not available.

### Hypercholesterolemia

Self-reported prevalence of hypercholesterolemia increased considerably between 1997 and 2007 (table 4) and this increase was further confirmed by multivariate analysis (table 5). Women, subjects over 45 years, with higher education or presenting with overweight or obesity had higher odds of reporting being hypercholesterolemic (table 5).

**Table 4:** trends in self-reported prevalence and management of hypercholesterolemia in the Swiss population, 1997 - 2007

	1997	2002	2007
Sum of weights	5,564,776	5,647,472	5,784,057
Screening	86.5	94.6	93.8
Prevalence	11.9	14.7	17.4
Treatment *	18.5	32.2	38.8
Control **	52.9	-	75.1

Results are expressed as weighted percentage. *, among subjects reporting being hypercholesterolemic; **, among treated subjects. -, data not available.

**Table 5:** multivariate analysis of the trends in self-reported prevalence and management of hypercholesterolemia in the Swiss population, 1997 - 2007

	Prevalence	Treatment*	Control**	Screening
Surveys				
1997	1 (ref.)	1 (ref.)	1 (ref.)	1 (ref.)
2002	1.26 [1.25 - 1.27]	2.21 [2.19 - 2.22]	-	2.77 [2.75 - 2.78]
2007	1.52 [1.51 - 1.53]	2.80 [2.78 - 2.82]	2.59 [2.55 - 2.63]	2.48 [2.47 - 2.49]
Gender				
Woman	1 (ref.)	1 (ref.)	1 (ref.)	1 (ref.)
Man	0.77 [0.76 - 0.78]	0.68 [0.67 - 0.69]	1.19 [1.18 - 1.21]	0.95 [0.95 - 0.96]
Age groups				
18-44	1 (ref.)	1 (ref.)	1 (ref.)	1 (ref.)
45-64	3.53 [3.52 - 3.55]	4.11 [4.07 - 4.15]	0.96 [0.93 - 0.98]	0.74 [0.74 - 0.75]
≥ 65	5.11 [5.09 - 5.13]	10.3 [10.2 - 10.4]	1.17 [1.14 - 1.20]	1.03 [1.02 - 1.04]
Nationality				
Swiss	1 (ref.)	1 (ref.)	1 (ref.)	1 (ref.)
Other	1.02 [1.01 - 1.03]	1.02 [1.01 - 1.03]	0.74 [0.72 - 0.75]	1.01 [1.01 - 1.01]
Education				
Low	1 (ref.)	1 (ref.)	1 (ref.)	1 (ref.)
Medium	1.09 [1.08 - 1.10]	0.96 [0.95 - 0.97]	1.15 [1.13 - 1.17]	0.85 [0.84 - 0.85]
High	1.24 [1.23 - 1.25]	0.86 [0.85 - 0.87]	1.58 [1.54 - 1.61]	0.69 [0.68 - 0.69]
BMI classes				
Normal	1 (ref.)	1 (ref.)	1 (ref.)	1 (ref.)
Overweight	1.46 [1.45 - 1.47]	1.41 [1.40 - 1.42]	1.09 [1.08 - 1.10]	0.95 [0.95 - 0.96]
Obesity	1.67 [1.66 - 1.68]	1.82 [1.80 - 1.83]	1.04 [1.02 - 1.06]	1.03 [1.02 - 1.03]

Results are expressed as multivariate-adjusted odds ratio and [95% confidence interval]. *, among subjects with reported hypercholesterolemia; **, among treated subjects.-, data not available.

Self-reported hypolipidemic drug treatment increased between 1997 and 2007 (table 4); multivariate analysis showed women, older subjects, subjects with a higher education or presenting with overweight or obesity to have higher odds of being treated (table 5). In 2007, 99.1% of hypolipidemic drug treatment was prescribed by the doctor and daily medication use was reported by 94.8% of treated subjects. The self-reported prevalence of controlled hypercholesterolemia increased (table 4); on multivariate analysis, women, subjects over 45 years, subjects with a medium and high education had a higher odds ratio, while foreigners had a lower odds ratio of reporting being adequately controlled (table 5). Conversely, the self-reported prevalence of uncontrolled and untreated hypercholesterolemia remained stable (table 4). Hypercholesterolemia screening increased (table 4); on multivariate analysis, a higher odds ratio of being screened was found for foreigners, subjects aged over 45, and in overweight or obese subjects, while women, subjects with a medium and a high education had a lower odds ratio of being screened (table 5).

### Diabetes

Self-reported prevalence of diabetes increased between 1997 and 2007 (table 6), a finding confirmed by multivariate analysis (table 7) which also showed men and subjects with increasing age or BMI to have a higher odds ratio, while subjects with middle or high education had a lower odds ratio of reporting being diabetic. Self-reported prevalence of diabetes treatment increased (table 6); multivariate analysis showed men, subjects aged over 45 or presenting with overweight or obesity to have a higher odds ratio, while foreigners had a lower odds ratio of being treated (table 7). Self-reported diabetes control also increased and the self-reported prevalence of uncontrolled and untreated diabetes decreased (table 6); multivariate analysis showed subjects aged 45-64 years, presenting with overweight or obesity or foreigners to have a lower odds ratio, while high educated subjects had a higher odds ratio of being controlled (table 7). Finally, diabetes screening increased during the study period (table 6) and multivariate analysis showed foreigners, subjects aged over 45, overweight or obese to have a higher odds ratio, while men and subjects with medium or high education to have a lower odds ratio of being screened (table 7).

**Table 6:** trends in self-reported prevalence and management of diabetes in the Swiss population, 1997 - 2007

	1997	2002	2007
Sum of weights	5,564,776	5,647,472	5,784,057
Diabetes (%)			
Screening	87.4	94.9	94.1
Prevalence	3.3	3.7	4.8
Treatment (drug) *	50.0	-	53.3
Control **	50.5	-	65.5

Results are expressed as weighted percentage. *, among subjects reporting being diabetic; **, among treated subjects. -, data not available.

**Table 7:** multivariate analysis of the trends in self-reported prevalence and management of diabetes in the Swiss population, 1997 - 2007

	Prevalence	Treatment *	Control **	Screening
Surveys				
1997	1 (ref.)	1 (ref.)	1 (ref.)	1 (ref.)
2002	1.10 [1.09 - 1.10]	-	-	2.68 [2.67 - 2.69]
2007	1.49 [1.48 - 1.50]	1.16 [1.15 - 1.18]	1.92 [1.88 - 1.95]	2.43 [2.42 - 2.45]
Gender				
Woman	1 (ref.)	1 (ref.)	1 (ref.)	1 (ref.)
Man	1.20 [1.19 - 1.20]	1.24 [1.23 - 1.26]	0.91 [0.90 - 0.93]	1.04 [1.04 - 1.04]
Age groups				
18-44	1 (ref.)	1 (ref.)	1 (ref.)	1 (ref.)
45-64	2.96 [2.94 - 2.98]	2.78 [2.73 - 2.84]	0.62 [0.60 - 0.65]	0.86 [0.86 - 0.87]
≥ 65	6.78 [6.73 - 6.83]	5.23 [5.13 - 5.34]	0.79 [0.76 - 0.82]	1.19 [1.19 - 1.20]
Nationality				
Swiss	1 (ref.)	1 (ref.)	1 (ref.)	1 (ref.)
Other	0.95 [0.95 - 0.96]	0.74 [0.73 - 0.76]	0.51 [0.50 - 0.52]	1.01 [1.00 - 1.01]
Education				
Low	1 (ref.)	1 (ref.)	1 (ref.)	1 (ref.)
Medium	0.79 [0.79 - 0.80]	1.46 [1.43 - 1.48]	1.13 [1.10 - 1.15]	0.87 [0.87 - 0.88]
High	0.75 [0.75 - 0.76]	1.00 [0.98 - 1.02]	1.86 [1.81 - 1.92]	0.64 [0.64 - 0.65]
BMI classes				
Normal	1 (ref.)	1 (ref.)	1 (ref.)	1 (ref.)
Overweight	1.64 [1.63 - 1.65]	1.63 [1.61 - 1.66]	1.15 [1.12 - 1.17]	0.95 [0.94 - 0.95]
Obesity	3.71 [3.68 - 3.73]	2.24 [2.20 - 2.27]	0.84 [0.82 - 0.86]	1.02 [1.01 - 1.03]

Results are expressed as multivariate-adjusted odds ratio and [95% confidence interval]. *, among subjects with reported diabetes; **, among treated subjects. -, data not available.

## Discussion

Since the MONICA study in the nineties [3] and the Bus Santé study in Geneva [4], there has been little information on trends of hypertension, hypercholesterolemia and diabetes in Switzerland. The data from the Swiss National Health Surveys thus provide important information regarding the self-reported prevalence and management of those cardiovascular risk factors in the Swiss population. As the sampling frame covers about 90% of Swiss households and the participation rate was relatively high for all studies, this study is a good reflect of the Swiss situation. The fact that the weighted and unweighted results were quite similar also suggests the absence of important bias.

### Hypertension

Prevalence of self-reported hypertension increased between 1997 and 2007 and was comparable to those reported using measured data by US [9] and German [10] studies and with other studies using self-reported data (table 8). This increase could be due either to an increase in the true prevalence of hypertension, to a more widespread screening, or both. The second hypothesis might be more likely, as the prevalence of subjects reporting having their blood pressure measured during the previous 12 months also increased during this period, a finding already reported in the literature [11]. Another likely determinant is decrease in the thresholds to define hypertension from ≥ 160/95 mmHg in 1993 [3] to ≥ 140/90 mmHg afterwards. Still, self-reported prevalence rates are probably underestimated, as a recent study conducted in Lausanne has shown that less than two thirds of hypertensive subjects are actually aware of their condition [12].

**Table 8:** trends in self-reported prevalence of cardiovascular risk factors in Switzerland and in other countries

	Switzerland	Spain	Greece	USA	France
Hypertension					
1997	22.1	11.4		24.4	
1999				25.4	
2001		14.5			
2002	22.4		20.1		
2003		14.5			
2006			25.7		
2007	24.2				
Dyslipidaemia					
1997	11.9	8.2		26.6	
1999				27.7	
2001		11.0			
2002	14.7		17.5		
2003		10.5			
2006			22.3		
2007	17.4				
Diabetes					
1997	3.3	5.0		6.5	8.5
1999				7.1	
2001		5.6			
2002	3.7		8.7		
2003		5.9			
2006			10.3		
2007	4.8				

References: Switzerland 1, current study; Spain, [21]; Greece, [28]; USA, [29]; France, [18].

A higher prevalence of reported hypertension was found among subjects aged over 45 years or presenting with overweight or obesity. Those findings are in agreement with the literature [9,13,14] and might be due to an increased screening with age or because of the presence of other risk factors [15]. Conversely, foreigners had a lower self-reported prevalence of hypertension, and this could not be attributed to a lower screening frequency or to differences in age or BMI status. Possible explanations include differences in dietary or genetic background, but further studies are needed to better assess this point. The self-reported prevalence of hypertension was also inversely related with educational level, a finding in agreement with the literature [16]. This finding might be related to a better lifestyle, namely regarding dietary salt intake, although data from the Geneva study showed no improvement in salt intake in the general population [17].

Self-reported treatment of hypertension increased during the study period, suggesting an improvement in the management of this risk factor. Still, in 2007, only six out of ten hypertensive subjects indicated they were on antihypertensive treatment. Although the remaining 40% might be under nonpharmacological antihypertensive measures such as diet or specific lifestyle modifications, our findings suggest that there is still room for improvement regarding pharmacological management of hypertension, a finding reported previously [12].

In agreement with objectively measured data from the US [9,13] and France [18], an increase in self-reported control of hypertension was found for the period 1997-2007. This increase might be related to an improvement in antihypertensive treatment, namely the appearance of more potent and new antihypertensive drugs, and/or an improvement of subject's compliance. Still, our results are probably overestimated because some treated subjects might report being controlled just because they are taking antihypertensive drugs. Indeed, a previous study conducted in Lausanne showed that a consistent fraction of treated hypertensive subjects actually presented with high blood pressure levels [12]. Hence, it is likely that the true prevalence of controlled hypertension in Switzerland might actually be lower. Nevertheless, the fact that the self-reported prevalence of uncontrolled and untreated hypertension also decreased suggests that the overall management of hypertension in the Swiss population is improving.

### Hypercholesterolemia

Self-reported prevalence of hypercholesterolemia was within values published for other countries which used self-reported data (table 8), but lower than the values obtained in a smaller Swiss population-based study using objectively measured data (table 9). Still, and in agreement with previous Swiss [4], French [18] and German [10] studies based on objectively measured data and with studies using self-reported data, the self-reported prevalence of hypercholesterolemia increased between 1997 and 2007. As for hypertension, possible explanations include a true increase in the prevalence of hypercholesterolemia, an increase in screening, a decrease in the threshold values to define hypercholesterolemia [19] or a mixture of them. Interestingly, cholesterol screening increased considerably during the study period, and the prevalence of subjects reporting having their blood cholesterol levels assessed during the previous 12 months was actually higher than other studies [11]. Still, in 2007, the self-reported prevalence of hypercholesterolemia in Switzerland was lower than the USA [11] or France [18]. Two explanations are possible, i.e. the prevalence of hypercholesterolemia being indeed lower in Switzerland, or a lower screening by Swiss GPs. Indeed, it has been shown that only 75% of Swiss physicians consider that screening for high cholesterol is very important, versus 93% for blood pressure [20]. Those differences could partly explain the lower percentage of self-reported hypercholesterolemia relative to hypertension.

**Table 9:** comparison of prevalences or hypertension and hypercholesterolaemia based on self-reported and measured data for subjects aged 35-75, Switzerland

	Switzerland 2002	Switzerland 2007	CoLaus 2003-6
Hypertension			
Prevalence	26.2	27.5	36.0
Treatment *	54.7	62.4	78.0
Control **	-	83.0	48.0
Hypercholesterolaemia			
Prevalence	18.6	22.4	29.0
Treatment *	32.7	40.4	40.0
Control **	-	77.1	58.0

Results are expressed as percentage. *, among subjects with the selected risk factor; **, among treated subjects. CoLaus data from [12] for hypertension and from [23] for hypercholesterolemia.

A higher self-reported prevalence of hypercholesterolemia was found among subjects aged over 45 years, with high education or presenting with overweight or obesity in agreement with other studies [16,18] but not with others [21]. Still, our results suggest that, contrary to hypertension, a higher education is related to a higher self-reported prevalence of hypercholesterolemia. This higher self-reported prevalence is not due to higher screening rates among highly educated subjects, as their odds of being screened were significantly lower (table 4). A possible explanation is the fact that highly educated subjects know better their medical situation [22], but again further studies are needed to better assess this point.

The self reported hypolipidemic treatment doubled during the study period, in line with other French [18] and German [10] studies. Nevertheless, in 2007, only four out of ten Swiss patients who had been told they presented with hypercholesterolemia reported being treated, a value similar to the one reported in the CoLaus study [23] (table 9). Although diet has been shown to lower cholesterol levels [24], it is unlikely that 60% of patients diagnosed with hypercholesterolemia are on a diet alone. Hence, and as for hypertension, our findings suggest that there is room for improvement regarding pharmacological management of hypercholesterolemia.

An increase in self-reported control of hypercholesterolemia was found, a finding also found in other countries [18,25]. Two hypotheses are possible, i.e. an improvement in hypolipidemic drugs and/or subject's compliance. Again, these results are certainly overestimated, either because the subjects believed they were controlled just because they were treated, or because their GP considered them as treated despite borderline high values [26].

### Diabetes

The self-reported prevalence of diabetes increased during period 1997-2007. Still, in 2007, the self-reported prevalence was lower than reported for France [18] or the US [27], probably due to the self-reported (instead of objectively measured) diabetic status. Still, comparing our data with self-reported data from other countries [18,21,28,29] led to similar conclusions (table 8). Possible explanations include the relatively low prevalence of obesity in Switzerland [30,31] albeit other factors might be at play. Interestingly, the increase in the self-reported prevalence of diabetes persisted after adjustment for overweight and obesity, suggesting that other factors might intervene [32], namely a better screening. Indeed, the prevalence of subjects reporting having their blood glucose assessed the previous 12 months increased between 1997 and 2007, a finding in agreement with other studies [33,34].

Also in agreement with the literature [32], a higher self-reported prevalence of diabetes was found among men, subjects aged over 45 years or presenting with overweight or obesity. Similarly, and as reported previously [16,32], a lower prevalence of self-reported diabetes was found among subjects with high educational level. High educated subjects could have more financial means to adapt their lifestyle, e.g., to buy higher quality food [35] or exercise more, thus preventing the occurrence of diabetes. They could better know their health state despite less screened, but further studies are needed to better assess this point.

Self reported antidiabetic treatment increased, a trend also reported for France [18] and Italy [36]. Still and again, in 2007, only half of the subjects diagnosed with diabetes reported being treated, and, as for hypercholesterolemia, it is rather unlikely that the remaining half was only on diet. Overall, our data indicate that, in Switzerland, many diabetic subjects are probably undertreated, and that further efforts should be made to implement (non) pharmacological treatment.

The increase in self-reported diabetic control found in this study has also been reported elsewhere [25]. This improvement is probably due to a change in therapies and/or an improvement of the subject's compliance. Still, in 2007, one third of treated diabetic subjects reported having high glycaemia, and again this figure is certainly underestimated because many treated subjects believed they are controlled simply due to the fact they receive a drug. Nevertheless, the fact that the prevalence of uncontrolled and untreated diabetes also decreased suggests that the overall management of diabetes in the Swiss population is improving.

### Limitations

First, and as indicated previously, the self-reporting of the cardiovascular risk factors might underestimate the real prevalence in the population, as it can be inferred from the results of table 9. Still, it represents the result of the screening done by doctors and health professionals and has been used in other studies for the assessment of trends [21,28,29,37]. Further, it has been shown that self-reported data on cardiovascular risk factors is valid and can be used to assess prevalence rates in most cases [38,39]. Second, increasing rates occurred mainly between 2002 and 2007, when the sample becomes much more educated, raising the issue of a possible selection bias, more educated participants tending to respond more easily. The presence of other unmeasured confounders such as changes in dietary intake could also influence results. Since other unmeasured predictors of disease treatment and control were likely to change along with education, the trends in treatment and control are thus likely to be biased away from the null. Still, in the absence of another nationally representative sample, this study provides the best estimates regarding self-reported prevalence and management of cardiovascular risk factors. Third, the fact that the unweighted and weighted estimates are similar does not remove the potential for response bias. Still, the weighting procedure gives some strata which are less represented in the sample (i.e. young males) a higher weight, thus partially reducing this bias. It should be noted that some studies only standardized on age [29] or even made no adjustment [28], while in this study weighting included gender, age, geographical location and nationality [8]. Forth, although several studies conducted in the USA [40,41] indicate a high level of undiagnosed hypertension and hypercholesterolemia among uninsured subjects, this is rather unlikely to occur in Switzerland as all subjects living in Switzerland have a health insurance (federal law 832.10 of march 18^th^, 1994, available at http://www.admin.ch/ch/f/rs/c832_10.html). Still, as a nontrivial percentage of subjects with hypertension, dyslipidemia or diabetes might be unaware of their status [12,42], our prevalence estimates might be underestimated. Finally, no information was available regarding nonpharmacological treatment of cardiovascular risk factors, so it was not possible to assess the percentage of subjects not treated with drugs but with other nonpharmacological measures.

## Conclusion

In Switzerland, self-reported prevalence of hypertension, hypercholesterolemia and diabetes have increased between 1997 and 2007. Management and screening have improved, but further improvements can still be achieved as over one third of subjects with reported CV RFs are not treated.

## Conflict of Interest Statement

The authors hereby indicate no conflict of interest

## Authors' contributions

DE performed the statistical analysis and wrote most of the manuscript. PMV designed the study (data analysis), obtained the data, performed some statistical analyses and wrote part of the manuscript. FP and PV participated in the study design and coordination and revised the manuscript. All authors read and approved the final manuscript.

## Pre-publication history

The pre-publication history for this paper can be accessed here:

http://www.biomedcentral.com/1471-2458/11/114/prepub

## Supplementary Material

Additional file 1 **Supplementary tables**. Click here for file
