# Supplementary material for: Trends in self-reported prevalence and management of hypertension, hypercholesterolemia and diabetes in Swiss adults, 1997-2007
Source: BMC Public Health. 2011 Feb 18;11:114. doi: 10.1186/1471-2458-11-114 (PMC3051907; doi:10.1186/1471-2458-11-114)
Supplement: Additional file 1 — Supplementary tables. [file 1471-2458-11-114-S1.DOC]

# Supplementary tables

Supplementary table 1**:** characteristics of the samples.

|  | **1997** | **2002** | **2007** |
| --- | --- | --- | --- |
| Sample size | 12,474 | 18,908 | 17,879 |
| Women (%) | 6937 (55.6) | 10345 (54.7) | 9862 (55.2) |
| Age classes (%) |  |  |  |
| 18-44 years | 6400 (52.6) | 8295 (45.4) | 7447 (43.6) |
| 45-64 years | 3616 (27.7) | 6357 (32.2) | 6066 (32.0) |
| ≥ 65 years | 2458 (19.7) | 4256 (22.5) | 4366 (24.4) |
| Swiss nationality (%) | 10,513 (84.3) | 16,614 (87.9) | 15,565 (87.1) |
| Educational level (%) |  |  |  |
| Low ^§^ | 2747 (22.0) | 3635 (19.2) | 2471 (13.8) |
| Middle ^§§^ | 7577 (60.7) | 12,082 (63.9) | 10,523 (58.9) |
| High ^§§§^ | 2150 (17.3) | 3191 (16.9) | 4885 (27.3) |
| BMI classes (%) |  |  |  |
| Normal | 8150 (65.3) | 11569 (61.2) | 10826 (60.5) |
| Overweight | 3450 (27.7) | 5749 (30.4) | 5465 (30.6) |
| Obese | 874 (7.0) | 1590 (8.4) | 1588 (8.9) |
| BMI [kg/m^2^] | 24.0 ± 4.0 | 24.4 ± 4.1 | 24.5 ± 4.1 |
| Age [years] | 46.7 ± 17.6 | 49.6 ± 17.1 | 50.4 ± 17.6 |

Results are expressed as number of subjects and (percentage) and average ± standard deviation. **^§^** no education completed + first level (primary school). **^§§^** lower + upper secondary level. **^§§§^** tertiary level + other education after secondary level.

Supplementary t**able 2:** trends in self-reported prevalence and management of hypertension in the Swiss population, 1997 – 2007.

|  | **1997** | **2002** | **2007** |
| --- | --- | --- | --- |
| Sample size | 12,474 | 18,908 | 17,879 |
| Screening | 10,927 (87.6) | 17,945 (94.9) | 16,973 (94.9) |
| Prevalence | 2775 (22.3) | 4616 (24.4) | 4785 (26.8) |
| Treatment * | 1451 (52.3) | 2534 (54.9) | 3063 (64.0) |
| Control ** | 824 (56.8) | - | 2516 (82.1) |

Results are expressed as percentage. *, among subjects reporting being hypertensive (i.e. for 1997: 1451/2775=52.3%); **, among subjects reporting being treated (i.e. for 1997: 824/1474=52.3%). -, data not available.

Supplementary t**able 3:** multivariate analysis of the trends in self-reported prevalence and management of hypertension in the Swiss population, 1997 – 2007.

|  | **Prevalence** | **Treatment*** | **Control**** | | **Screening** |
| --- | --- | --- | --- | --- | --- |
| Surveys |  |  |  |  | |
| 1997 | 1 (ref.) | 1 (ref.) | 1 (ref.) | 1 (ref.) | |
| 2002 | 1.00 [0.94 - 1.06] | 0.96 [0.87 - 1.07] | - | | 2.55 [2.35 - 2.78] |
| 2007 | 1.11 [1.04 - 1.17] | 1.34 [1.20 - 1.49] | 3.31 [2.87 - 3.82] | 2.54 [2.33 - 2.77] | |
| Gender |  |  |  | |  |
| Woman | 1 (ref.) | 1 (ref.) | 1 (ref.) | 1 (ref.) | |
| Man | 1.02 [0.97 - 1.06] | 0.85 [0.78 - 0.93] | 0.87 [0.75 - 1.01] | | 0.77 [0.72 - 0.83] |
| Age groups |  |  |  |  | |
| 18-44 | 1 (ref.) | 1 (ref.) | 1 (ref.) | | 1 (ref.) |
| 45-64 | 2.84 [2.68 - 3.01] | 4.92 [4.35 - 5.57] | 2.01 [1.52 - 2.66] | 1.42 [1.30 - 1.54] | |
| ≥ 65 | 7.25 [6.83 - 7.70] | 13.7 [12.0 - 15.5] | 2.03 [1.55 - 2.66] | | 2.40 [2.14 - 2.69] |
| Nationality |  |  |  |  | |
| Swiss | 1 (ref.) | 1 (ref.) | 1 (ref.) | | 1 (ref.) |
| Other | 0.91 [0.85 - 0.98] | 0.89 [0.78 - 1.02] | 0.64 [0.51 - 0.80] | 1.11 [1.00 - 1.23] | |
| Education |  |  |  | |  |
| Low | 1 (ref.) | 1 (ref.) | 1 (ref.) | 1 (ref.) | |
| Medium | 0.93 [0.87 - 0.98] | 0.99 [0.90 - 1.10] | 1.30 [1.10 - 1.53] | | 0.96 [0.86 - 1.06] |
| High | 0.87 [0.81 - 0.94] | 1.03 [0.90 - 1.18] | 1.68 [1.32 - 2.13] | 0.88 [0.78 - 1.00] | |
| BMI classes |  |  |  | |  |
| Normal | 1 (ref.) | 1 (ref.) | 1 (ref.) | 1 (ref.) | |
| Overweight | 1.99 [1.89 - 2.09] | 1.39 [1.27 - 1.52] | 1.13 [0.97 - 1.32] | | 1.23 [1.13 - 1.33] |
| Obesity | 4.07 [3.78 - 4.38] | 1.99 [1.76 - 2.24] | 1.10 [0.91 - 1.34] | 1.43 [1.23 - 1.67] | |

Results are expressed as multivariate-adjusted odds ratio and [95% confidence interval]. *, among subjects with reported hypertension; **, among treated subjects. -, data not available.

Supplementary t**able 4:** trends in self-reported prevalence and management of hypercholesterolemia in the Swiss population, 1997 – 2007.

|  | **1997** | **2002** | **2007** |
| --- | --- | --- | --- |
| Sample size | 12,474 | 18,908 | 17,879 |
| Screening | 10,729 (86.0) | 17,807 (94.2) | 16,720 (93.5) |
| Prevalence | 1535 (12.3) | 3021 (16.0) | 3489 (19.5) |
| Treatment * | 278 (18.1) | 989 (32.7) | 1460 (41.9) |
| Control ** | 145 (52.2) | - | 1120 (76.7) |

Results are expressed as number of subjects and (percentage). *, among subjects reporting being hypercholesterolemic (i.e. for 1997: 278/1535=18.1%); **, among subjects reporting being treated (i.e. for 1997: 145/278=52.2%). -, data not available.

Supplementary t**able 5:** multivariate analysis of the trends in self-reported prevalence and management of hypercholesterolemia in the Swiss population, 1997 – 2007.

|  | **Prevalence** | **Treatment** | **Control** | **Screening** |
| --- | --- | --- | --- | --- |
| Surveys |  |  |  |  |
| 1997 | 1 (ref.) | 1 (ref.) | 1 (ref.) | 1 (ref.) |
| 2002 | 1.24 [1.16 - 1.32] | 2.18 [1.86 - 2.55] |  | 2.67 [2.46 - 2.89] |
| 2007 | 1.53 [1.43 - 1.64] | 3.10 [2.66 - 3.62] | 2.82 [2.15 - 3.70] | 2.43 [2.25 - 2.63] |
| Gender |  |  |  |  |
| Woman | 1 (ref.) | 1 (ref.) | 1 (ref.) | 1 (ref.) |
| Man | 1.25 [1.18 - 1.31] | 1.42 [1.27 - 1.58] | 1.17 [0.92 - 1.49] | 0.95 [0.89 - 1.02] |
| Age groups |  |  |  |  |
| 18-44 | 1 (ref.) | 1 (ref.) | 1 (ref.) | 1 (ref.) |
| 45-64 | 3.35 [3.14 - 3.58] | 3.67 [3.05 - 4.42] | 1.37 [0.85 - 2.2] | 0.79 [0.73 - 0.85] |
| ≥ 65 | 4.87 [4.54 - 5.22] | 8.88 [7.36 - 10.71] | 1.71 [1.07 - 2.74] | 1.07 [0.97 - 1.17] |
| Nationality |  |  |  |  |
| Swiss | 1 (ref.) | 1 (ref.) | 1 (ref.) | 1 (ref.) |
| Other | 1.04 [0.96 - 1.12] | 0.93 [0.78 - 1.10] | 0.76 [0.53 - 1.08] | 0.95 [0.87 - 1.05] |
| Education |  |  |  |  |
| Low | 1 (ref.) | 1 (ref.) | 1 (ref.) | 1 (ref.) |
| Medium | 1.05 [0.98 - 1.13] | 0.86 [0.75 - 0.98] | 1.10 [0.83 - 1.45] | 0.88 [0.80 - 0.96] |
| High | 1.19 [1.09 - 1.29] | 0.78 [0.66 - 0.92] | 1.63 [1.13 - 2.36] | 0.75 [0.67 - 0.84] |
| BMI classes |  |  |  |  |
| Normal | 1 (ref.) | 1 (ref.) | 1 (ref.) | 1 (ref.) |
| Overweight | 1.45 [1.37 - 1.53] | 1.43 [1.28 - 1.60] | 1.06 [0.83 - 1.34] | 0.98 [0.91 - 1.05] |
| Obesity | 1.60 [1.47 - 1.74] | 1.87 [1.60 - 2.19] | 0.94 [0.68 - 1.30] | 1.08 [0.95 - 1.23] |

Results are expressed as multivariate-adjusted odds ratio and [95% confidence interval]. *, among subjects with reported hypercholesterolemia; **, among treated subjects.-, data not available.

Supplementary t**able 6:** trends in self-reported prevalence and management of diabetes in the Swiss population, 1997 – 2007.

|  | **1997** | **2002** | **2007** |
| --- | --- | --- | --- |
| Sample size | 12,474 | 18,908 | 17,879 |
| Screening | 10,841 (86.9) | 17,855 (94.4) | 16,768 (93.8) |
| Prevalence | 409 (3.3) | 809 (4.3) | 946 (5.3) |
| Treatment (drug) * | 200 (48.9) | - | 556 (57.1) |
| Control ** | 100 (50.0) | - | 366 (65.8) |

Results are expressed as number of subjects and (percentage). *, among subjects reporting being diabetic (i.e. for 1997: 200/409=48.9%); **, among subjects reporting being treated (i.e. for 1997: 100/200=50%); -, data not available.

Supplementary t**able 7:** multivariate analysis of the trends in self-reported prevalence and management of diabetes in the Swiss population, 1997 – 2007.

|  | **Prevalence** | **Treatment *** | **Control **** | **Screening** |
| --- | --- | --- | --- | --- |
| Surveys |  |  |  |  |
| 1997 | 1 (ref.) | 1 (ref.) | 1 (ref.) | 1 (ref.) |
| 2002 | 1.19 [1.05 - 1.34] | - | - | 2.56 [2.36 - 2.78] |
| 2007 | 1.51 [1.34 - 1.71] | 1.33 [1.04 - 1.70] | 1.91 [1.36 - 2.68] | 2.34 [2.16 - 2.54] |
| Gender |  |  |  |  |
| Woman | 1 (ref.) | 1 (ref.) | 1 (ref.) | 1 (ref.) |
| Man | 1.25 [1.14 - 1.37] | 1.35 [1.06 - 1.71] | 0.99 [0.72 - 1.37] | 1.03 [0.96 - 1.11] |
| Age groups |  |  |  |  |
| 18-44 | 1 (ref.) | 1 (ref.) | 1 (ref.) | 1 (ref.) |
| 45-64 | 3.01 [2.63 - 3.45] | 2.91 [1.96 - 4.31] | 0.62 [0.31 - 1.25] | 0.91 [0.84 - 0.98] |
| ≥ 65 | 6.35 [5.55 - 7.26] | 4.83 [3.30 - 7.06] | 0.82 [0.42 - 1.61] | 1.24 [1.13 - 1.37] |
| Nationality |  |  |  |  |
| Swiss | 1 (ref.) | 1 (ref.) | 1 (ref.) | 1 (ref.) |
| Other | 0.99 [0.86 - 1.14] | 0.86 [0.60 - 1.22] | 0.58 [0.36 - 0.95] | 0.97 [0.88 - 1.07] |
| Education |  |  |  |  |
| Low | 1 (ref.) | 1 (ref.) | 1 (ref.) | 1 (ref.) |
| Medium | 0.77 [0.69 - 0.85] | 1.11 [0.85 - 1.45] | 1.07 [0.75 - 1.52] | 0.88 [0.80 - 0.97] |
| High | 0.74 [0.64 - 0.85] | 0.98 [0.69 - 1.40] | 1.90 [1.14 - 3.17] | 0.72 [0.64 - 0.80] |
| BMI classes |  |  |  |  |
| Normal | 1 (ref.) | 1 (ref.) | 1 (ref.) | 1 (ref.) |
| Overweight | 1.60 [1.44 - 1.77] | 1.68 [1.30 - 2.18] | 0.91 [0.63 - 1.33] | 0.98 [0.91 - 1.06] |
| Obesity | 3.75 [3.33 - 4.22] | 2.45 [1.81 - 3.32] | 0.89 [0.59 - 1.35] | 1.11 [0.97 - 1.27] |

Results are expressed as multivariate-adjusted odds ratio and [95% confidence interval]. *, among subjects with reported diabetes; **, among treated subjects. -, data not available.
